# Association between neutropenia and survival to nab-paclitaxel and gemcitabine in patients with metastatic pancreatic cancer

**DOI:** 10.1038/s41598-020-76465-9

**Published:** 2020-11-06

**Authors:** Giandomenico Roviello, Monica Ramello, Martina Catalano, Alberto D’Angelo, Raffaele Conca, Silvia Gasperoni, Lorenzo Dreoni, Roberto Petrioli, Anna Ianza, Stefania Nobili, Michele Aieta, Enrico Mini

**Affiliations:** 1 Department of Health Sciences, University of Florence, viale Pieraccini, 6, 50139 Florence, Italy; 2 Oncology Unit, Department of Medical, Surgical, and Health Sciences, University of Trieste, Piazza Ospitale, Trieste, Italy; 3 School of Human Health Sciences, University of Florence, Largo Brambilla 3, 50134 Florence, Italy; 4 Department of Biology and Biochemistry, University of Bath, Bath, BA2 7AY UK; 5 Division of Medical Oncology, Department of Onco-Hematology, IRCCS-CROB, Referral Cancer Center of Basilicata, via Padre Pio 1, 85028 Rionero, Vulture, PZ Italy; 6 Translational Oncology Unit, University Hospital Careggi, Firenze, Toscana Italy; 7 Department of Medicine, Surgery and Neurosciences, Medical Oncology Unit, University of Siena, Viale Bracci-Policlinico “Le Scotte”, 53100 Siena, Italy

**Keywords:** Cancer, Cancer therapy, Chemotherapy

## Abstract

Neutropenia is a common side effect associated with nab-paclitaxel gemcitabine (Nab-Gem) therapy. We retrospectively investigated the association between neutropenia induced by first-line Nab-Gem and survival in metastatic pancreatic carcinoma patients. Metastatic pancreatic patients treated with first-line Nab-Gem were included in this retrospective analysis. Neutropenia was categorized using the National Cancer Institute Common Toxicity Criteria scale. Outcome measures were overall survival (OS), progression-free survival (PFS) and response rate. 115 patients were analyzed. Median PFS was 7 months (95% CI 5–8) for patients with grade ≥ 3 neutropenia and 6 months (95% CI 5–6) for patients with grade < 3 neutropenia [p = 0.08; hazard ratio (HR 0.68)]. Median OS was 13 months (95% CI 10–18) for patients with grade ≥ 3 neutropenia and 10 months (95% CI 8–13) for patients with grade < 3 neutropenia (p = 0.04; HR 0.44). In multivariate analysis, the occurrence of grade ≥ 3 neutropenia showed a statistically significant association with OS (HR 0.62; 95% CI 0.09–0.86; p = 0.05). Nab-Gem-induced neutropenia is associated with longer survival in metastatic pancreatic cancer patients.

## Introduction

Pancreatic Cancer is the 12th most frequent cancer worldwide and, given its poor prognosis, the 4th cause of cancer-related death in Western Countries^1^. Generally, radiotherapy and surgery can be considered at early-stage or locally advanced disease^2,3^; unfortunately, despite recent developments in diagnosis, most patients can show asymptomatic advanced or metastatic disease. For these patients, clinical trial enrolment^4^ or chemotherapy can be offered, whit the latter the current standard of care with an estimated 5-year survival rate of 5%, can be considered^5^. Based on this scenario, there is a strong need to find predictive factors of response to chemotherapy.

Taxanes such as paclitaxel and docetaxel are chemotherapeutic agents that mainly suppress microtubules dynamics and stabilise GDP-bound tubulin in the microtubule^6^. In particular, paclitaxel is formulated in the Cremophor EL (polyoxyl 35 castor oil) solvent, essentially a polyethoxylated oil which results in hypersensitivity and anaphylactic reactions^7,8^. Therefore, paclitaxel is also formulated with steroids and a histamine H2 receptor blocker-based premedication to reduce hypersensitivity reactions^7,8^. Nanoparticle entailing albumin-bound (nab) paclitaxel provides a solvent-free formulation of paclitaxel, minimizing the risk of hypersensitivity^9,10^. In 2013, the phase III MPACT trial showed a significant survival benefit (1-year OS rate of 35% versus 22%) of the nab-paclitaxel/gemcitabine (Nab-Gem) combination compared to gemcitabine monotherapy, with 8.5 versus 6.7 months overall survival (OS) and 0.72 hazard ratio (HR) for death in favour of Nab-Gem group^11^. The progression-free survival (PFS) was longer in the experimental arm (5.5 months for the Nab-Gem group and 3.7 months for the gemcitabine group, HR 0.69) and the response rate (RR) was 23% for Nab-Gem arm versus 7% for the control arm. Based on these results, Nab-Gem is widely considered a valid option for patients with metastatic pancreatic cancer as first-line chemotherapy. Additional data in support of Nab-Gem efficacy for pancreatic cancer comes from Real-world experiences^12–14^. In these studies, the median OS ranged from 9.2 to 10.9 months and median PFS ranged from 5.2 to 6.7 months in favour of patients treated with Nab-Gem.

Although the use of Nab-Gem combinational therapy is consolidated in daily clinical practice, only limited data discuss the possible predictive factor of Nab-Gem efficacy in pancreatic cancer. However, different studies reported a positive correlation between taxanes-based neutropenia and the increase of OS and PFS^15–17^, suggesting that a lower number of neutrophils (neutropenia) might be considered as a predictive biomaker of efficacy in patients treated with taxanes as mono or combinational therapy. Therefore, this study aims to investigate the correlation between the development of grade ≥ 3 neutropenia and survival of first-line Nab-Gem in patients with metastatic pancreatic cancer.

## Materials and methods

### Eligibility criteria

We performed a retrospective study involving 4 different Italian oncological centres across the North, Central and South of Italy. Patients were diagnosed with metastatic pancreatic carcinoma by either histological or cytological biopsy. All patients presented an Eastern Cooperative Oncology Group (ECOG) PS of ≤ 2, adequate haematological function (defined as the number of white blood cells > 4000/μL and absolute neutrophil count > 1500/μL, haemoglobin ≥ 9 g/dL, platelets > 100.000/mm^3^), and satisfactory renal and hepatic function (defined as serum bilirubin level at or below the upper limit of normal range). Patients who underwent surgery or adjuvant treatments (chemotherapy or radiation therapy) were evaluated only if the aforementioned treatments occurred more than 6 weeks before the start of Nab-Gem therapy. All included patients had at least one cycle of treatment completed. Exclusion criteria included serious cardiovascular problems (i.e., ejection fraction < 40%, myocardial infarction) or infections. All patients gave their written consent and the protocol was approved by the Institutional Review Board for clinical trials of Tuscany: section AREA VASTA CENTRO, number:14565_oss.

### Treatment plan and response assessments

The treatment consisted of nab-paclitaxel (125 mg/m^2^) plus gemcitabine (1000 mg/m^2^) administered on days 1, 8, 15 every 28 days until disease progression or unacceptable toxicity. Second or additional therapy lines were administered according to the single centre experience. Patients received antiemetic medication at the beginning of each treatment cycle. Chemotherapeutic cycles were administered only with absolute neutrophil count > 1500/μL, haemoglobin ≥ 9 g/dL and platelets > 100.000/mm^3^. Analgesic drugs were administered at adequate doses to provide optimal pain control. Clinical, radiological and biochemical pre-treatment assessments were performed within 2 weeks from beginning of treatment while blood tests before every single drug administration. Tumour burden response was assessed with RECIST 1.1 criteria^18^ every three months or earlier when clinically required.

### Neutropenia assessment

Neutropenia was assessed by the National Cancer Institute Common Toxicity Criteria toxicity scale, version 4.2^19^. Grade 1 was defined with the neutrophil count from the lower limit of normal (LLN) to 1500/mm^3^; grade 2 with the neutrophil count from < 1500 to 1000/mm^3^; grade 3 with the neutrophil count from < 1000 to 500/mm^3^; grade 4 with neutrophil count < 500/mm^3^. Dose modification, delay and drug-discontinuation related to neutropenia or other adverse events (AE) were performed according to the drug sheet. Granulocyte-colony stimulating factor (G-CSF) was administered according to the local clinical practice, although no cytokine prophylactic treatment was administered.

### Statistical analysis

This study aimed to evaluate whether the development of grade ≥ 3 neutropenia positively correlates with efficacy and survival of patients with metastatic pancreatic cancer treated with Nab-Gem as first-line treatment. For this purpose, patients were split into two groups according to the development of grade ≥ 3 neutropenia as a cut-off. Patient and tumour characteristics plus treatment data were collected as frequency, percentage of categorical variables, median with 95% confidence interval and range (for continuous variables). PFS was evaluated from the Nab-Gem regimen start until progression of disease or death as well as OS was evaluated from the Nab-Gem regimen start until death. Kaplan–Meier method with log-rank test was performed to analyse PFS and OS in relation to the development of grade ≥ 3 neutropenia. Cox regression model was used to evaluate the prognostic role of neutropenia and other clinical and/or pathological variables. Statistical analysis was performed using STATA software with a statistical significance threshold agreed upon p < 0.05.

### Ethical approval

All procedures performed in studies involving human participants were in accordance with the ethical standards of the institutional and/or national research committee and with the 1964 Helsinki declaration and its later amendments or comparable ethical standards.

### Informed consent

Informed consent was obtained from all individual participants included in the study.

## Results

### Patient characteristics

From January 2015 to December 2018 a total of 115 patients diagnosed with metastatic pancreatic cancer and treated with first-line Nab-Gem were retrospectively investigated^20^. Of these, 26 patients (22.6%) developed grade ≥ 3 neutropenia and 89 (77,4%) developed grade < 3 neutropenia during treatment. The median age was 67.5 years (range 51–84) for grade ≥ 3 neutropenia group while 65 years (range 50–83) for grade < 3 neutropenia group (p = 0.7). 10 (38.5%) patients and 28 (31.5%) patients were over 70 years in grade ≥ 3 neutropenia group and grade < 3 neutropenia group (p = 0.5), respectively. Sex distribution was similar between the two groups (p = 0.5). A larger percentage of patients within the grade < 3 neutropenia group reported an ECOG = 1 when compared with patients within grade ≥ 3 neutropenia group (55.1% vs 50%; p = 0.4). The most common metastatic sites were liver and lung with liver metastasis more common in the grade ≥ 3 neutropenia group (65.4% vs 52.8%; p = 0.1) whereas lung metastases more common in the grade < 3 neutropenia group (26.8% vs 15.4%; p = 0.1). Concerning previous treatments, most patients in the grade < 3 neutropenia group underwent surgery and radiotherapy (27%; 10.1%; vs 15.4%; 3.8%) while biliary stent was previously placed in the 38.5% of patients in the grade ≥ 3 neutropenia group vs 31.5% of patients in the grade < 3 neutropenia group (p = 0.5). Basal carbohydrate antigen 19–9 (CA 19-9) levels showed no differences between the two groups (p = 0.2). Baseline patient characteristics are summarized in Table 1.

**Table 1:** Patient characteristics.

	All patients (N = 115)	Neutropenia < 3 (N = 89)	Neutropenia ≥ 3 (N = 26)	p
**Age, years**				
Median	65	65	67.5	0.7
Range	50–84	50–83	51–84	
≥ 70	38 (33%)	28 (31.5%)	10 (38.5%)	0.5
**ECOG PS**				
0	53 (46.1)	40 (44.9%)	13 (50%)	0.4
1	62 (53.9%)	49 (55.1%)	13 (50%)	
**Sex**				
Male	61 (53%)	47 (52.8%)	14 (53.8%)	0.5
Female	54 (47%)	42 (47.2%)	12 (46.2%)	
**Stage at diagnosis**				
i–iii	30 (26.1%)	24 (26.8%)	6 (23.1%)	0.6
iv	85 (73.9%)	65 (73.2%)	20 (76.9%)	
**Site of metastatic disease**				
Liver	64 (55.6%)	47 (52.8%)	17 (65.4%)	0.1
Lung	28 (24.3%)	24 (26.8%)	4 (15.4%)	0.1
Peritoneum	16 (13.9%)	13 (14.6%)	3 (11.5%)	0.4
Others	11 (9.6%)	8 (9%)	3 (11.5%)	0.3
**Number of metastatic sites**				
1–2	69 (60%)	51 (57.3%)	16 (61.5%)	0.3
≥ 3	46 (40%)	38 (42.7%)	10 (38.5%)	
**Carbohydrate antigen 19-9 (U/ml)**				
Median	659	659	649	0.2
Range	0.8–182,922	0.8–182,922	0.8–15,126	
**Previous treatment**				
Radiation therapy	10 (8.7%)	9 (10.1%)	1 (3.8%)	0.3
Surgery	28 (24.3%)	24 (27%)	4 (15.4%)	0.2
Biliary stent	38 (33%)	28 (31.5%)	10 (38.5%)	0.5
Chemotherapy	0	0	0	
**Pain**				
Yes	48 (41.7%)	38 (42.7%)	10 (38.5%)	0.7

### Neutropenia and clinical outcome

Twenty-six patients (22.6% of the entire population) developed grade ≥ 3 neutropenia during treatment and, among these, 8 (30.8%) developed grade 4 neutropenia; however, 3 (11.5%) patients discontinued Nab-Gem therapy due to neutropenia severity. Among those patients with grade ≥ 3 neutropenia, treatment delay occurred in 10 (34.5%) patients with an average delay of 7 days. A dose reduction was required for 14 (53.8%) patients.

**Figure 1:**
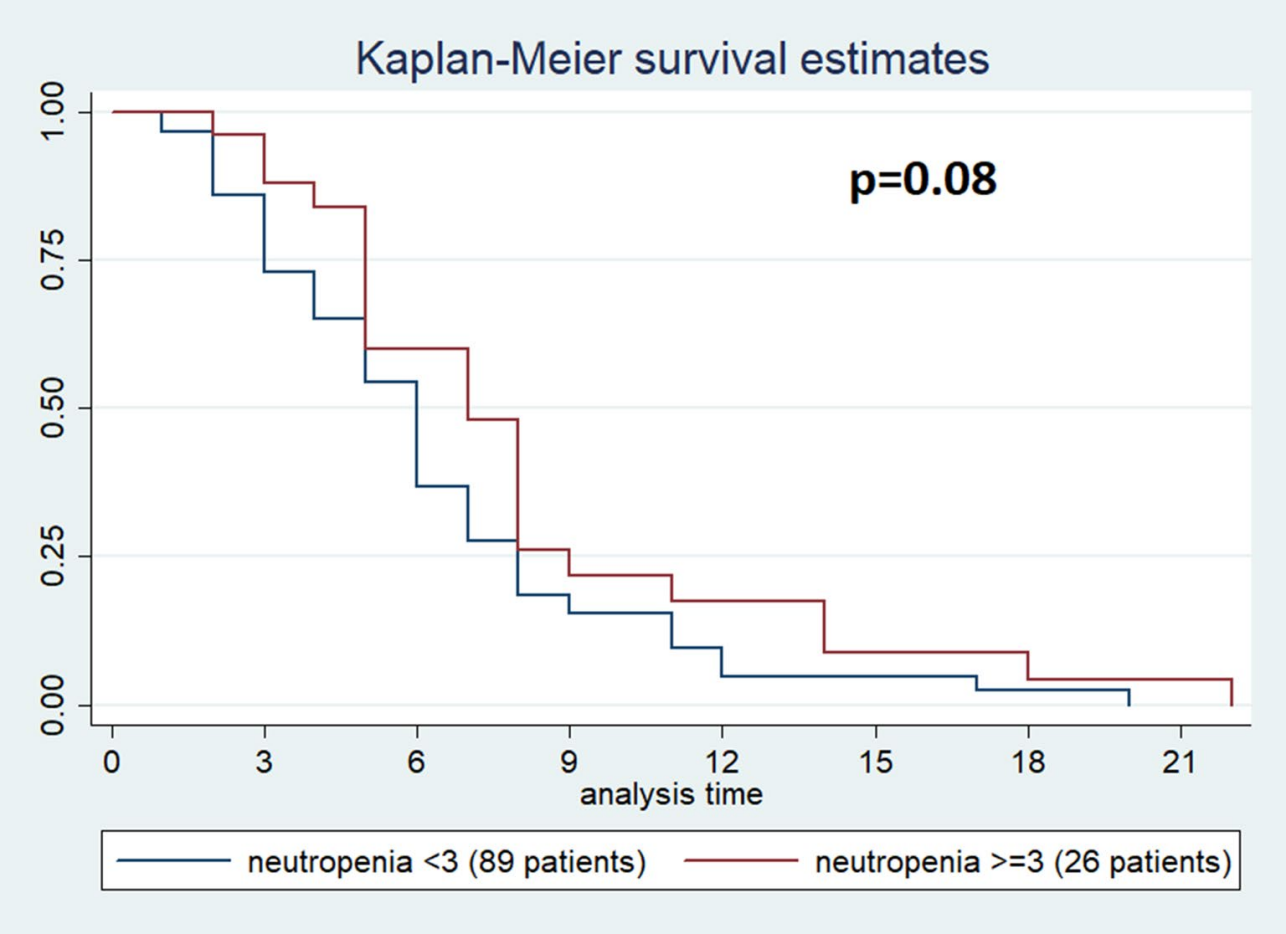
Estimated PFS for Nab-Gem in patients with grade ≥ 3 neutropenia (red) or without (blue).

**Figure 2:**
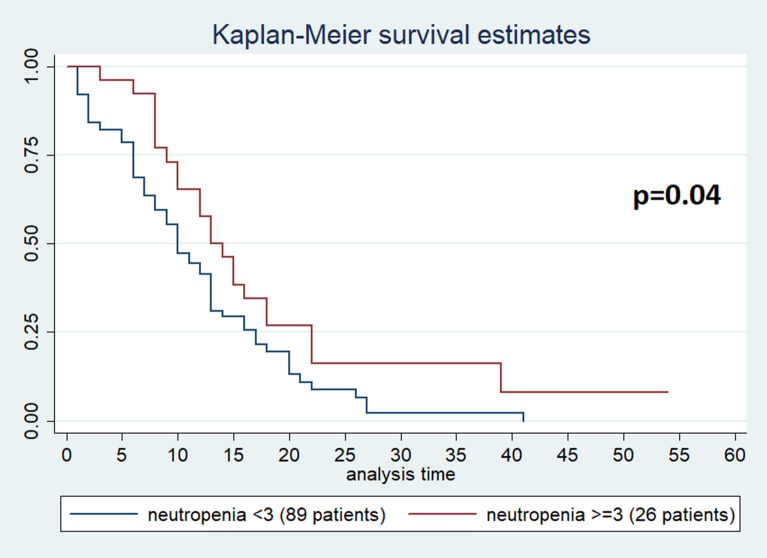
Estimated OS for Nab-Gem in patients with grade ≥ 3 neutropenia (red) or without (blue).

No febrile neutropenia occurred and 25 (21.9% of the entire population) patients received G-CSF; among these, 11 (12.5%) patients developed grade < 3 neutropenia whereas 14 (53.8%) patients developed grade ≥ 3 (p = 0.01). Concerning efficacy data, after a median follow-up of 10 months, median PFS was 6 months [95% CI (5–7 months)] while median OS was 11 months [95% CI 11 (9–13 months)]; no complete responses (CR) were observed and disease control rate (DCR) was 64.3% (74 out of 115 patients) among all patients (Table 2). In particular, patients who developed grade ≥ 3 neutropenia had a median PFS of 7 months [95% CI (5–8 months)] compared to the PFS of 6 months [95% CI (5–6 months)] for patients with grade < 3 neutropenia (p = 0.08) (Fig. 1). While grade ≥ 3 neutropenia patients had a median OS of 13 months [95% CI (10–18 months)], patients with grade < 3 neutropenia reported and OS of 10 months [95% CI (8–13 months)] (p = 0.04) (Fig. 2). DCR was achieved by 84% of patients with grade ≥ 3 neutropenia and by 58% of patients with no severe neutropenia (p = 0.06) (Table 2). A total of 47 (40.91% of the entire population) patients were treated with second-line therapy, 36 (40.4%) from the group of patients with grade < 3 neutropenia and 11 (42.3%) from the group of patients with grade ≥ 3 neutropenia (irinotecan-based chemotherapy was the preferred second-line regimen). The results of the univariate analysis for OS show that (Table 3) age ≥ 70, ECOG-PS = 1, number of metastatic sites baseline ≥ 3 and CA 19–9 ≥ 659 U/ml were found negative prognostic factors, whereas previous surgery and grade ≥ 3 neutropenia (HR 0.62, 95% CI 0.38–1.0, p = 0.05) were found to be significantly positive prognostic factors. The multivariate analysis confirms that age ≥ 70, number of metastatic sites, CA 19-9 and grade ≥ 3 neutropenia were independently associated with OS (Table 4). The univariate and multivariate analysis for PFS are reported in Tables 3, 4.

**Table 2:** Best response, PFS and OS according to neutropenia grade.

	All patients (N = 115)	Neutropenia < 3 (N = 89)	Neutropenia ≥ 3 (N = 26)	p
PR	44 (38.3%)	29 (32.6%)	15 (57.7%)	0.06
SD	30 (26.1%)	23 (25.8%)	7 (26.9%)	
DCR (PR + SD)	74 (64.3%)	52 (58.4%)	22 (84.6%)	
PD	32 (27.8%)	29 (32.6%)	3 (11.5%)	
NE	9 (7.8%)	8 (9%)	1 (3.8%)	
**PFS**				
M-months	6	6	7	0.08
(95% IC)	(5–7)	(5–6)	(5–8)	
**OS**				
M-months	11	10	13	**0.04**
95% IC	(9–13)	(8–13)	(10–18)	
**Cycles**				
Median	5	4	6	0.9
Range	1–17	1–17	1–17	
GCF-prophylaxis	25 (21.9%)	11 (12.5%)	14 (53.8%)	**0.01**

Bold values are statistically significant.

*N* Number, *PR* partial response, *SD* stable disease, *DCR* disease control rate, *PD* progression disease, *NE* not evaluable, *median* Median, *PFS* progression free survival, *OS* overall survival.

**Table 3:** Univariate analysis for PFS and OS.

	HR	IC 95%	p
**Progression-free survival**			
Age ≥ 70	1.44	0.93–2.24	0.1
ECOG PS (1 vs 0)	1.17	0.78–1.77	0.4
Sex (male vs female)	1.08	0.71–1.64	0.7
N. of metastatic sites ≥ 3	**3.85**	**2.06–7.20**	**0.001**
Carbohydrate antigen 19-9 ≥ 659 U/ml	**1.86**	**1.23–2.83**	**0.003**
Previous radiation therapy	0.63	0.29–1.37	0.2
Previous Surgery	0.76	0.47–1.26	0.2
Previous Biliary stent	0.75	0.48–1.17	0.2
Pain present	**1.51**	**1–2.31**	**0.05**
Neutropenia ≥ 3	0.68	0.42–1.09	0.1
**Overall survival**			
Age ≥ 70	**1.88**	**1.23–2.89**	**0.004**
ECOG PS (1 vs 0)	**1.52**	**1–2.31**	**0.05**
Sex (male vs female)	1.20	0.79–1.83	0.4
N. of metastatic sites ≥ 3	**3.91**	**2–7.63**	**< 0.001**
Carbohydrate antigen 19-9 ≥ 659 U/ml	**1.71**	**1.22–2.60**	**0.01**
Previous radiation therapy	0.47	0.19–1.16	0.1
Previous surgery	**0.58**	**0.35–0.99**	**0.04**
Previous Biliary stent	0.84	0.54–1.32	0.4
Pain present	1.50	0.98–2.29	0.06
Neutropenia ≥ 3	**0.62**	**0.38–1.0**	**0.05**

Bold values are statistically significant.

**Table 4:** Multivariate analysis for PFS and OS.

	HR	IC 95%	p
**Progression-free survival**			
N. of metastatic sites ≥ 3	**2.73**	**1.41–5.27**	**0.003**
Carbohydrate antigen 19–9 ≥ 659 U/ml	**1.67**	**1.08–2.57**	**0.02**
Neutropenia ≥ 3	**0.76**	**0.46–1.25**	**0.29**
Pain present	1.30	0.84–2.01	0.23
**Overall survival**			
Age ≥ 70	**1.66**	**1.01–2.59**	**0.02**
ECOG PS (1 vs 0)	1.32	0.85–2.04	0.2
N. of metastatic sites ≥ 3	**1.91**	**1.22–4.02**	**0.04**
Carbohydrate antigen 19–9 ≥ 659 U/ml	**1.65**	**1.05–2.60**	**0.03**
Previous surgery	0.61	0.36–1.01	0.08
Neutropenia ≥ 3	**0.61**	**0.37–1.00**	**0.05**

Bold values are statistically significant.

## Discussion

Pancreatic cancer accounts for the fourth cause of cancer-related deaths worldwide; unfortunately, the prognosis of metastatic pancreatic cancer is very scarce with an approximately 5% overall 5-year survival rate^1^. From 2013, the combination of Nab-Gem is a possible treatment as first-line therapy for patients with metastatic pancreatic cancer^11^. Neutropenia is a common side effect associated with Nab-Gem as reported by the MPACT phase III trial, where nearly 40% of patients developed grade ≥ 3 neutropenia^11^. In addition, two studies evaluating the efficacy and safety of Nab-Gem treatment for metastatic pancreatic cancer in a ‘real-world’ showed that grade 3 and 4 neutropenia have been observed in 21% and 23% of patients, respectively^12,13^. In our current study, approximately 23% of patients developed grade ≥ 3 neutropenia and, despite the limited patients’ group size, these patients had longer OS (Figs. 1, 2) than those with grade < 3 neutropenia. Moreover, a potential positive correlation between the grade of neutropenia and PFS or DCR has been observed. In 2018, a Chinese study by Chen et al. investigated 134 patients correlating the timing of early-onset chemotherapy-induced neutropenia (defined as the development of neutropenia by the end of cycle 2) and prognosis in patients with advanced pancreatic cancer undergoing gemcitabine/gemcitabine-based chemotherapy^21^. The authors speculated that early-onset neutropenia predicts longer survival. However, we report that 69 (51.5%) patients received Nab-Gem in the aforementioned study and, unlike our study, the authors defined neutropenia cut-off at the lowest point with a neutrophil count of < 2.0 × 10^9^/L. On the contrary, we investigated different grades of neutropenia according to the National Cancer Institute Common Toxicity Criteria, offering a more precise evaluation of neutropenia. Also, we evaluated only patients treated with Nab-Gem and metastatic disease whereas Chen et al. investigated patients with locally advanced disease who received either gemcitabine monotherapy or other gemcitabine-based chemotherapy regimens.

The prognostic role of neutropenia might be explained in multiple ways: firstly, systemic inflammation actively supports tumour initiation, promotion and progression; secondly, myelosuppression in severe neutropenic patients might lead to a significant reduction of myeloid-derived suppressing cells (MDSCs), resulting in the suppression of CD4+ T cells action against late-stage malignancies^22^.

The correlation between neutropenia and taxanes efficacy has been previously demonstrated. For example, two independent pharmacokinetic analysis of RAINBOW phase-III trial including Western and East Asian patients treated with paclitaxel plus ramucirumab showed that grade ≥ 3 neutropenia significantly correlated with the efficacy of the combination regimen^15,16^. Similarly, other retrospective analysis confirmed the correlation between paclitaxel plus ramucirumab-induced neutropenia and treatment-efficacy as well as longer survival in patients with metastatic gastric cancer^23^. Also docetaxel, an additional taxane chemotherapeutic agent, has been evaluated for a potential correlation between its efficacy and neutropenia. In 2018, a retrospective study investigated the association between chemotherapy-induced neutropenia and survival in metastatic castration-resistant prostate cancer (CRPC) patients receiving first-line docetaxel^17^. Eighty patients were analysed showing an 0.36 h for grade 2–3 neutropenia and 0.19 h for grade 4 neutropenia when compared to grade 0–1 neutropenia. The author hypothesized that docetaxel-induced neutropenia is associated with longer survival of individuals diagnosed with metastatic castration-resistant prostate cancer. The aforementioned studies suggest that the higher-grade neutropenia caused by taxane-based chemotherapy, potentially lead to longer survival. In line with these data, our study seems to confirm the role of grade ≥ 3 neutropenia as a predictor of Nab-Gem therapy efficacy.

The FOLFIRINOX regimen, namely the association of 5-fluorouracil, irinotecan-and oxaliplatin, is also adopted for the treatment of metastatic pancreatic cancer as first-line therapy. In 2011, the PRODIGE4/ACCORD11 trial showed that FOLFIRINOX led to longer survival rates when compared to gemcitabine monotherapy^24^. In 2018, a study investigated the effect of severe neutropenia on clinical outcomes in advanced pancreatic cancer patients who received modified FOLFIRINOX^25^. This study assessed a total of 51 patients treated from January 2014 until May 2018. The result of this study showed that the median OS was significantly longer in patients with severe neutropenia than in those with lower neutropenia grade (1 or 2).

In our study, none of the patients experienced febrile neutropenia although 25 (21.9%) of these received G-CSF—most of them (53.8%) belonged to the group of patients who developed grade ≥ 3 neutropenia. In 2017, the results of a systematic literature review and meta-analysis showed a modest survival increase for patients undergoing intensified chemotherapy with G-CSF support for solid tumours and lymphoma, compared with those receiving standard chemotherapy^26^. However, no conclusive data are available for patients with metastatic pancreatic cancer and prospective studies are required to determine whether G-CSF has any effect on survival.

Optimal second-line chemotherapy following Nab-Gem regimen and subsequent progression of disease is unclear. In this study, a similar percentage of patients were treated with a second-line therapy (40.4% vs 43.2%). In 2019, a prospective study^27^ showed that second-line fluoropyrimidine-based regimens after Nab-Gem are achievable, reporting manageable toxicity as well as a longer survival for patients administered with irinotecan-combination regimen. In our study, irinotecan-based chemotherapy was the preferred second-line regimen for both groups.

Finally, this study presented several limitations that we must report: firstly, the retrospective source of data; secondly, the limited number of patients assessed, together with the lack of a control arm. However, few data are currently available on predictive factors of efficacy for patients treated with Nab-Gem.

## Conclusions

Although it is arduous to draw a defined conclusion, we report the robust correlation between Nab-gem therapy response and occurrence of grade ≥ 3 neutropenia. In conclusion, Nab-Gem-induced neutropenia might be a prognostic factor of survival in patients with metastatic pancreatic cancer treated with the combination of Nab-Gem. Prospective large-scale trials are needed to further confirm this result.

## Abbreviations

Nab-Gem: Nab-paclitaxel gemcitabine
OS: Overall survival
PFS: Progression-free survival
HR: Hazard ratio
RR: Response rate
ECOG: Eastern Cooperative Oncology Group
AE: Adverse event
G-CSF: Granulocyte-colony stimulating factor
CR: Complete responses
DCR: Disease control rate
MDSCs: Myeloid-derived suppressing cells
CRPC: Castration-resistant prostate cancer
